# Regional moderate hyperthermia for mild-to-moderate COVID-19 (TherMoCoV study): a randomized controlled trial

**DOI:** 10.3389/fmed.2023.1256197

**Published:** 2023-12-22

**Authors:** Javier Mancilla-Galindo, Ashuin Kammar-García, María de Lourdes Mendoza-Gertrudis, Javier Michael García Acosta, Yanira Saralee Nava Serrano, Oscar Santiago, Miriam Berenice Torres Vásquez, Daniela Martínez Martínez, Liliana Aline Fernández-Urrutia, Julio César Robledo Pascual, Iván Daniel Narváez Morales, Andrea Aida Velasco-Medina, Javier Mancilla-Ramírez, Ricardo Figueroa-Damián, Norma Galindo-Sevilla

**Affiliations:** ^1^División de Posgrado, Facultad de Medicina, Universidad Nacional Autónoma de México, Mexico City, Mexico; ^2^Dirección de Investigación, Instituto Nacional de Geriatría, Mexico City, Mexico; ^3^Servicio de Alergia e Inmunología Clínica, Hospital General de México, Mexico City, Mexico; ^4^Sección de Estudios de Posgrado e Investigación, Escuela Superior de Medicina, Instituto Politécnico Nacional, Mexico City, Mexico; ^5^Unidad Temporal COVID-19 Autónomo Hermanos Rodríguez, Instituto Mexicano del Seguro Social, Mexico City, Mexico; ^6^Departamento de Infectología e Inmunología, Instituto Nacional de Perinatología, Mexico City, Mexico; ^7^St. Luke Medical School of Alliant International University, Mexico City, Mexico; ^8^Hospital Regional de Alta Especialidad “Dr. Juan Graham Casasus”, Secretaría de Salud, Villahermosa, Mexico; ^9^Hospital de la Mujer, Secretaría de Salud, Mexico City, Mexico

**Keywords:** thermotherapy, non-oncologic applications of hyperthermia, COVID-19, SARS-CoV-2, hypoxemic respiratory failure

## Abstract

**Background:**

To prevent COVID-19 progression, low-cost alternatives that are available to all patients are needed. Diverse forms of thermotherapy have been proposed to prevent progression to severe/critical COVID-19.

**Objective:**

The aim of this study is to evaluate the efficacy and safety of local thermotherapy to prevent disease progression in hospitalized adult patients with mild-to-moderate COVID-19.

**Methods:**

A multicenter, open-label, parallel-group, randomized, adaptive trial is used to evaluate the efficacy and safety of local thermotherapy to prevent disease progression in hospitalized adult patients with mild-to-moderate COVID-19. Eligible hospitalized adult patients with symptoms of COVID-19 with ≤5 days from symptom onset, meeting criteria for mild or moderate COVID-19, were randomly assigned to the intervention consisting of local thermotherapy via an electric heat pad in the thorax (target temperature range 39.5–42°C) continuously for 90 min, twice daily, for 5 days, or standard care. The main outcome was the proportion of patients who progressed to severe-to-critical COVID-19 or death. Patients were randomized in a 1:1 ratio through a centralized computer-generated sequence of minimization with a random component of 20%. Participants and medical staff were not blinded to the intervention.

**Results:**

One-hundred and five participants (thermotherapy *n* = 54, control *n* = 51) with a median age of 53 (IQR: 41–64) years were included for analysis after the early cessation of recruitment due to the closure of all temporal COVID-19 units (target sample size = 274). The primary outcome of disease progression occurred in 31.4% (16/51) of patients in the control group vs. 25.9% (14/54) of those receiving thermotherapy (risk difference = 5.5%; 95%CI: −11.8–22.7, *p* = 0.54). Thermotherapy was well tolerated with a median total duration of thermotherapy of 900 (IQR: 877.5–900) min. Seven (13.7%) patients in the control group and seven (12.9%) in the thermotherapy group had at least one AE (*p* = 0.9), none of which were causally attributed to the intervention. No statistically significant differences in serum cytokines (IL-1β, IL-6, IL-8, IL-10, IL-17, and IFN-γ) were observed between day 5 and baseline among groups.

**Conclusion:**

Local thermotherapy was safe and well-tolerated. A non-statistically significant lower proportion of patients who experienced disease progression was found in the thermotherapy group compared to standard care. Local thermotherapy could be further studied as a strategy to prevent disease progression in ambulatory settings.

**Clinical Trial registration**: www.clinicaltrials.gov, identifier: NCT04363541.

## Introduction

Coronavirus disease (COVID-19), caused by the severe acute respiratory syndrome coronavirus 2 (SARS-CoV-2), remains a leading cause of illness and mortality worldwide. COVID-19 was the main cause of death in Mexico in the year 2021 (1) and the sixth cause of death in the general population in 2022 (2). Vaccination coverage against SARS-CoV-2 in Mexico was estimated to be 74% in 2021, according to a national seroprevalence study (3). On 22 September 2023, the Mexican Federal Commission for the Protection against Health Risks (COFEPRIS) announced a cease in the emergency use authorization of COVID-19 vaccines, which has caused concerns about a potential brand-specific vaccine shortage in Mexico (4).

Current challenges of COVID-19 disease include limited access to vaccines and medications (5), reduced diagnostic testing to assess the true remaining impact of the disease (6), and increased susceptibility of vulnerable populations (i.e., increased age, comorbidities, and social deprivation) to severe disease (6, 7). Numerous drugs have been shown to be safe and effective in treating patients with severe COVID-19 (i.e., corticosteroids, baricitinib, or IL-6 receptor blockers) (8), but few have been shown to prevent disease progression. Nirmatrelvir-ritonavir is the only drug with a strong recommendation to treat patients with mild COVID-19, while molnupiravir and remdesivir have weak or conditional recommendations in favor (8). Nonetheless, high prices and limited availability have limited their use, especially in low-middle-income settings (9). Thus, low-cost alternatives to prevent disease progression that are easily available to all patients are still needed. Thermal-based therapies have the potential to overcome the limitations of current medications against COVID-19 (10), and their study is relevant in the COVID-19 vaccine era since their hypothesized mechanisms of action are not virus-specific and could potentially be used against other respiratory viruses.

Hyperthermia (also thermotherapy) consists of the application of local heat through modalities such as radiofrequency, microwaves, hot water baths, sauna, lasers, magnetic fluids, and high-intensity focused ultrasound, which have been proposed as useful therapies for treating infectious diseases (11), including COVID-19 (12–15). Recently, core warming of COVID-19 patients under mechanical ventilation to a maximum temperature of 39.8°C was found to be feasible and safe in a pilot study (16). We previously reviewed the molecular and cellular mechanisms of increased susceptibility to heat by SARS-CoV-2, the enhanced immune response against the virus, and the clinical considerations of implementing thermotherapy-based interventions for the management of COVID-19 (10). Of note, thermotherapy-based interventions should be low-cost, widely accessible, targeted early on after infection (within the first 5 days after symptom onset), and aimed at preventing progression in patients with mild-to-moderate COVID-19.

Two randomized controlled studies to date have assessed a thermotherapy-based intervention (short-wave diathermy) in patients with moderate (17) and moderate-to-severe (18) COVID-19, establishing the safety of diathermy and promising efficacy in the treatment of COVID-19. In our study, we sought to evaluate a different modality of thermotherapy consisting of locally administered heat in the thorax through an electric pad. Therefore, the primary objective of this study was to evaluate the efficacy of local thermotherapy to prevent disease progression in hospitalized adult patients with mild-to-moderate COVID-19. Secondary objectives included assessment of safety, whole-blood cytokines and biomarkers, and other clinical outcomes.

## Methods

### Study design and setting

This was a multicenter, open-label, parallel-group, randomized, explanatory, adaptive trial to evaluate local thermotherapy in patients with mild-to-moderate COVID-19 to prevent disease progression. Patients were randomized in a 1:1 ratio to receive usual in-hospital care only (control group) or local thermotherapy for 5 days in addition to usual in-hospital care (intervention group) and followed up until day 28 after randomization. This study was approved by the ethics committees of *Dirección General de Calidad y Educación en Salud* (CEI-DGCES/2020:03.1) and *Instituto Nacional de Perinatología* (2020-1-19) of the Health Secretariat of Mexico and was prospectively registered (NCT04363541).

Three temporal COVID-19 units in Mexico City, Jalisco, and Tabasco recruited patients, but only the participants in Mexico City and Tabasco were included since the Jalisco center was closed after the inclusion of five patients who did not complete follow-up. This trial was adaptive since it considered the possibility of changes in the standard of care due to the rapid emergence of evidence on COVID-19 treatment. Nonetheless, changes in the standard of care did not occur during the study period (from 27 August 2020 to 23 August 2021) in these two centers. The study was stopped early after a recommendation from the independent trial monitoring committee since all temporal COVID-19 units were dismantled due to a decreasing number of COVID-19 cases.

Eligible participants were patients with symptoms of COVID-19 (fever, headache, cough, sore throat, myalgias, arthralgias, shortness of breath, anosmia, fatigue, diarrhea, vomiting, or conjunctivitis) who were admitted to hospital upon a compatible clinical presentation, meeting criteria for mild or moderate COVID-19 (Table 1) (19, 20), less than or equal to 5 days from symptom onset, and agreeing to participate in the study with randomization. Exclusion criteria included suspected or confirmed pregnancy at evaluation, severe decompensation of underlying diseases, prior diagnosis of COVID-19 with complete resolution of symptoms for at least 2 days, and patients meeting criteria for severe or critical COVID-19 (Table 1) at evaluation. Elimination criteria were withdrawal of consent to participate in the study, a requirement of ≥4 L/min of supplementary oxygen in the first 24 h of hospitalization (21), two subsequent negative SARS-CoV-2 diagnostic tests (RT-PCT and rapid antigen test) after enrollment, participants requesting to stop receiving the intervention before completing 5 days, and transfer to another medical unit within the first 5 days of participation in the study.

**Table 1:** COVID-19 severity definitions.

Mild COVID-19	With or without mild pneumonia. Peripheral oxygen arterial saturation (SpO_2_) greater than or equal to 94% (90% in Mexico City) at room temperature. Does not meet the criteria for moderate, severe, or critical COVID-19.
Moderate COVID-19	Patient with pneumonia and risk factors for disease progression; meeting all the following: Shortness of breath, SpO_2_ greater than or equal to 94% (90% in Mexico City) with a maximum 3 L/min of supplementary oxygen, does not meet the criteria for severe or critical COVID-19.
Severe COVID-19	≥1 of the following: tachypnea (≥30 breaths per minute), SpO_2_ lower than or equal to 93% (89% in Mexico City) with a maximum of 3 L/min of supplementary oxygen (patients requiring ≥4 L/min will be considered to have progressed to severe COVID-19), or PaO_2_/FiO_2_ ratio < 300.
Critical COVID-19	Patient with ARDS, shock, multiorgan failure, or any other condition requiring admission to an intensive care unit.

Classification criteria (19, 20) were adapted to match the definitions of progression used in the study used for the sample size calculation (21) and adjusted for the normal peripheral oxygen saturation values in Mexico City (2,240 m above sea level) (43–45). ARDS, acute respiratory distress syndrome; COVID-19, coronavirus disease; FiO_2_, fraction of inspired oxygen; PaO_2_, arterial pressure of oxygen; SpO_2_, peripheral arterial oxygen saturation.

### Intervention

The intervention consisted of local thermotherapy via an electric heat pad (30 × 40 cm) in the thorax continuously for 90 min, twice daily (every 12 h), for 5 days. The heat pads increased the external temperature regionally to 40.5°C (range: 39.5–42°C). Calibration of the devices was verified before application. Trained personnel applied the intervention and monitored vital signs and tolerance every 15 min. The intervention was interrupted if burns or other skin lesions occurred, regardless of being associated or not with the intervention, if the patient did not tolerate the intervention, or if any other severe adverse events occurred. Both the control and intervention groups received standard in-hospital care according to national guidelines. Standard of care consisted of usual in-hospital care, including but not limited to oxygen therapy, thromboprophylaxis, systemic corticosteroids upon supplementary oxygen requirement, and any other interventions prescribed by the treating medical team. Treating physicians were discouraged from prescribing treatments (including antivirals and other immunomodulatory agents) that were being studied for the management of COVID-19 and were not recommended in the national guidelines as part of the standard of care at that moment. The list of potential co-interventions was obtained from the ASHP list (22), and any prescription of selected medications available at the hospitals was recorded and described in the baseline characteristics of participants in the Results section.

### Outcomes

The primary outcome was a composite outcome of progression to severe COVID-19, critical COVID-19, or death (Table 1). The progression of the disease was monitored continuously during hospitalization by attending physicians not involved in the study. After hospital discharge, follow-up was made via telephone call on days 15 and 28.

Secondary outcomes were mortality at days 15 and 28, time to progression to severe and critical COVID-19 (days from symptom onset to progression), duration of hospitalization (days), clinical status in the ordinal scale at days 15 and 28 (23), time (days) to weaning from oxygen therapy according to modality (simple nasal cannula or face mask, non-rebreather mask, high-flow nasal cannula, non-invasive mechanical ventilation, and invasive mechanical ventilation), changes in National Early Warning Score 2 (NEWS-2) (24) with respect to baseline (days 1, 5, 15, and 28), proportion of patients requiring IMV and ICU admission and time-to-event, tolerance to the intervention (defined as the proportion of participants tolerating the intervention according to the number of uninterrupted thermotherapy sessions and minutes per session), adverse events (AEs) according to outcome, severity, and causality, and changes in serum cytokine levels and laboratory parameters (leukocytes, neutrophils, lymphocytes, albumin, lactate dehydrogenase, fibrinogen, D-dimer, C-reactive protein, procalcitonin, and erythrocyte sedimentation rate). Additional laboratory parameters (glucose, urea, blood urea nitrogen, creatinine, total bilirubin, direct bilirubin, indirect bilirubin, aspartate aminotransferase (AST), alanine aminotransferase (ALT), creatinine phosphokinase (CPK), hemoglobin, hematocrit, monocytes, platelets, neutrophil-to-lymphocyte ratio, international normalized ratio (INR), prothrombin time, and partial thromboplastin time) were also included as secondary outcomes.

The clinical status in the ordinal scale has been recommended as a measure of progression for COVID-19 trials (25). We used the ordinal scale developed by Wang et al. (23), which consists of the following seven categories:

1. not hospitalized, without limitations on daily activities;
2. not hospitalized, with limitations on daily activities;
3. hospitalized, not requiring supplementary oxygen;
4. hospitalized, requiring low-flow supplementary oxygen;
5. hospitalized, requiring supplementary oxygen with a high-flow nasal cannula or non-invasive ventilation;
6. hospitalized, under invasive mechanical ventilation or extracorporeal membrane oxygenation (ECMO); and
7. death.

AEs were classified according to the 2012 Mexican operative norm (NOM-220-SSA1-2012) as “any undesirable medical occurrence that can present during the investigational phase of a drug that may or may not be causally related to the drug,” according to outcome, severity, and causality (26). Based on the outcome, a severe AE was one that causes the death of the patient, puts the patient’s life at risk in the moment of occurrence, requires hospitalization or prolongs hospitalization, or causes persistent or significant disability; non-severe AEs were those not meeting the prior definition. The severity of an AE was consigned according to the Common Terminology Criteria for Adverse Events (CTCAE = v.5.0) (27) as mild (grade 1), moderate (grade 2), or severe (grades 3, 4, and 5).

The causality of AEs was classified as certain when they occurred after the administration of the intervention, could not be explained by the natural history of the disease or other diseases or drugs, and when the interruption of the intervention led to an evident clinical improvement response of the AE. Probable AEs were those meeting the above criteria, with an expectation that the clinical response to the interruption of the intervention was reasonable rather than evident, whereas possible AEs did not lead to clinical improvement, or this was not clear. Uncertain AEs were those in which the sequence of events led to an improbable causality of the AE, likely explained by the natural history of disease or concomitant diseases or drugs. Unclassifiable AEs were those that could not be classified as any of the above due to insufficient or contradictory information that could not be verified.

### Sample size

The estimation was calculated to find a reduction of 10% as the minimal clinically relevant difference in the proportion of patients who progressed from mild/moderate COVID-19 to severe/critical COVID-19 (13% according to Huang et al.) (21) with a statistical power of 80%, a two-sided significance level of 5%, and adjusted for an expected 20% losses during follow-up, yielding an estimated sample size of 274 patients (137 per group).

No stopping rules for efficacy endpoints were used to avoid overestimation of the benefits (28). All AEs of possible, probable, or certain causality were reported to the external monitoring committee. Stopping rules were based on the occurrence of at least one grade 4 or 5 AE of probable or certain causality.

### Randomization and blinding

Patients were randomized in a 1:1 ratio through a centralized computer-generated sequence of minimization with a random component of 20%. The automated assignment system was built with the open-code OxMaR software (29) in its Spanish version (30). Variables known to be associated with COVID-19 progression (31) were used for minimization: sex (male/female), age (cutoff of 50 years), body mass index (cutoff of 30 kg/m^2^), diabetes (yes/no), hypertension (yes/no), and supplementary oxygen at admission (yes/no). The allocation sequence was concealed from all researchers; only JMG (who had no role in the decision to include patients in the study) had access to it. The decision to randomize a patient was independently made at each center on a case-by-case basis. Participants and medical staff could not be blinded to the intervention since a medical device was employed. The data analyst (AKG) was blinded to the treatment groups.

### Determination of cytokine levels in peripheral blood

Whole-blood samples were collected through venipuncture at baseline and day 5 (days 15 and 28 were only collected for those patients who remained hospitalized) in lithium heparin tubes, after which they were centrifuged and preserved at −70°C with anti-protease medium until analysis using an ELISA immunoassay to quantify cytokine (IL-1β, IL-6, IL-8, IL-10, IL-17, and IFN-γ) levels (Supplementary Table S1).

### Statistical analysis

Intention-to-treat analyses were applied for primary and secondary outcomes. For the primary outcome of progression to severe COVID-19, critical COVID-19, or death at day 28, the proportions of patients who progressed by treatment group were compared with the chi-squared test, and the results were presented as frequencies with percentages (%). Cumulative probabilities of progression during follow-up were graphed (Kaplan–Meier estimator) by treatment group and compared with the log-rank test.

For the secondary endpoints of mortality at days 15 and 28, IMV requirement, ICU admission, clinical status at days 15 and 28, and the incidence of adverse events, we compared the proportions by chi-squared or Fisher’s exact test, and the results were presented as frequencies with percentages (%). The secondary outcomes of time to progression to severe and critical COVID-19, duration of hospitalization, time to weaning from oxygen therapy according to modality, time to requiring IMV, and ICU admission were compared using the Mann–Whitney U-test and presented as the median with interquartile range (IQR). The changes in NEWS-2, serum cytokine levels, and laboratory parameters with respect to baseline were compared using the Mann–Whitney U-test and presented as the median of the difference in each participant (Δ) with interquartile range (IQR).

For the changes in serum cytokine levels and laboratory parameters, we calculated the ratio of change as the percentage of change with respect to baseline, presented as the geometric mean. Box-and-whisker (1.5 times the IQR) plots were used to present changes in NEWS-2 score, peripheral blood cytokine levels, and peripheral blood inflammatory markers. The ratio of change was calculated for peripheral blood cytokines and inflammatory markers for days 5 and 15, both compared to baseline levels, and are presented as the geometrical mean for each variable.

Mortality risk was estimated for all patients at baseline by calculating the PH-COVID-19 risk score (31), which included eight predictors of mortality (age, sex, diabetes, chronic obstructive pulmonary disease, immunosuppression, hypertension, obesity, and chronic kidney disease) and was created and validated to be applied in the Mexican population. According to the calculated score, participants were categorized into low (−2 to 2 points), medium-low (3 to 5 points), medium (6 to 8 points), medium-high (9 to 15 points), and high (>15 points) risk categories.

**Figure 1:**
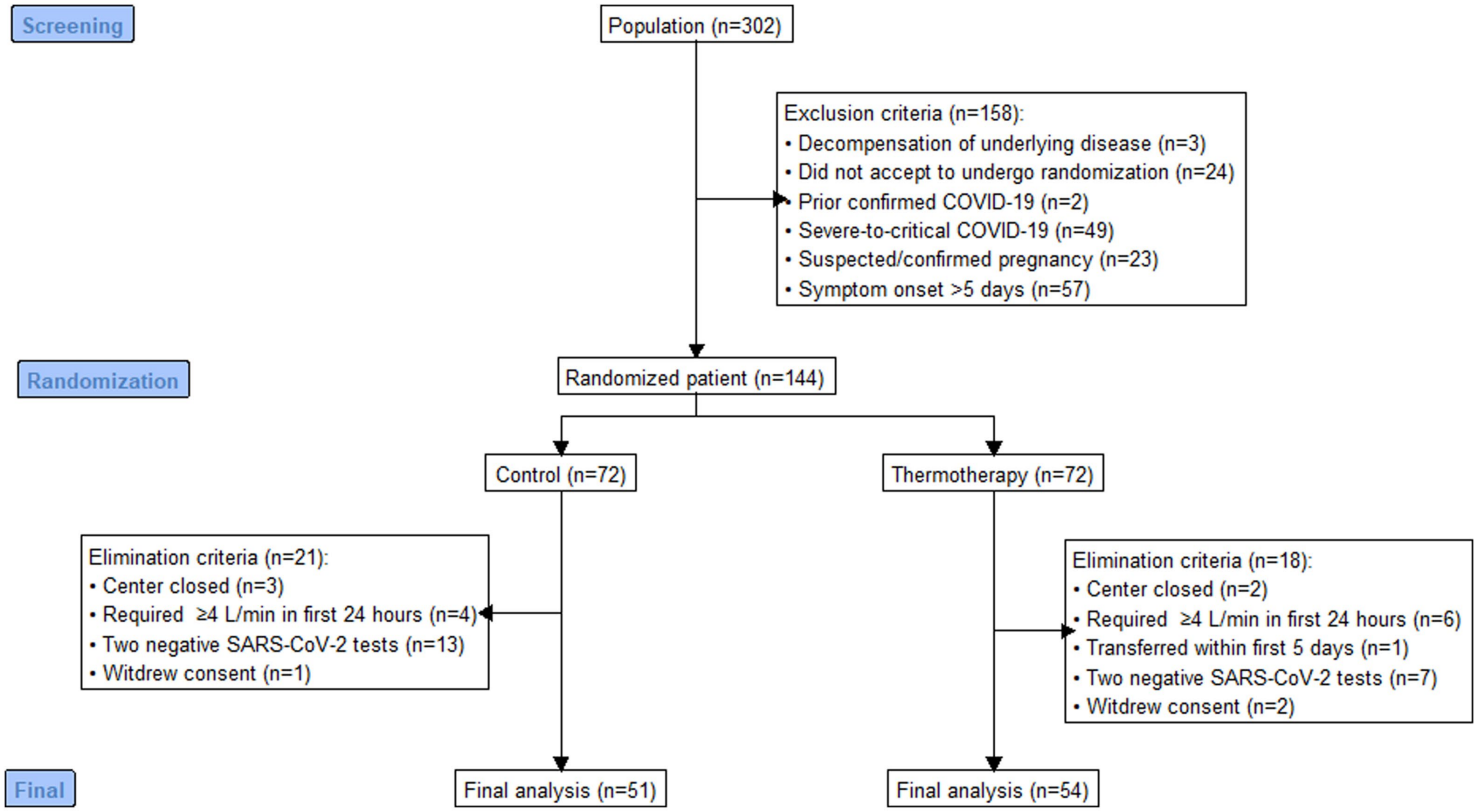
Flowchart of the participants.

Data imputation was not performed, except for the NEWS-2 score, for which the main analysis without data imputation is presented, alongside the analysis considering the latest NEWS-2 score registered for all patients. A *p*-value <0.05 was used to define statistical significance. All statistical analyses were performed in SPSS v.21. Data visualization was carried out in GraphPad v.9.1.1, except for Figure 1, which was built with the *consort* package (32) in R (33).

## Results

Out of 302 patients assessed for eligibility, 144 were randomized to thermotherapy (*n* = 72) or standard care (*n* = 72), and 105 participants (thermotherapy *n* = 54, control *n* = 51) were included for analysis (Figure 1). Reasons for exclusion and elimination of patients were pre-specified per protocol, except for five patients randomized in the Jalisco center, which was closed before patients completed follow-up. Out of all the included patients, 55.2% (*n* = 58) were women, with a median age of 53 (IQR: 41–64) years, admitted with predominantly moderate COVID-19 (81.9%), over mild COVID-19 (18.1%), and most had a *medium-high* (47.6%), *medium* (22.9%), or *medium-low* (18.1%) mortality risk score. The baseline characteristics of participants (Table 2) show a balanced distribution of variables used for minimization.

**Table 2:** Baseline characteristics of the participants.

	Total	Control	Thermotherapy
Number of patients	105	51	54
Sex, *n* (%)			
Female	58 (55.2)	28 (54.9)	30 (55.6)
Male	47 (44.8)	23 (45.1)	24 (44.4)
Age, years	53 (41–64)	52 (40–60)	55 (41.3–66.5)
Weight, Kg	75 (69.5–86)	78 (70–90)	75 (65.5–85)
Height, m	1.61 (1.56–1.68)	1.63 (1.56–1.70)	1.60 (1.54–1.66)
BMI	28.9 (26.1–33.5)	28.9 (25.8–31.9)	28.8 (26.2–34.6)
PH-COVID-19* risk categories, *n* (%)			
Low (−2 to 2 points)	8 (7.6)	3 (5.9)	5 (9.3)
Medium-low (3 to 5 points)	19 (18.1)	11 (21.6)	8 (14.8)
Medium (6 to 8 points),	24 (22.9)	11 (21.6)	13 (24.1)
Medium-high (9 to 15 points)	50 (47.6)	26 (51.0)	24 (44.4)
High (>15 points)	4 (3.8)	0 (0.0)	4 (7.4)
SBP, mmHg	118 (110–120)	120 (110–122)	110 (109.8–120)
DBP, mmHg	70 (70–80)	70 (70–80)	70 (66.5–80)
HR, bpm	79 (70–90)	80 (70–91)	77.5 (70–90)
RR, bpm	22 (20–24)	22 (20–23)	22 (21–24)
Temperature, °C	36.2 (36–36.6)	36.5 (36–36.7)	36 (36–36.5)
SpO_2_, %	94 (92–95)	94 (92–95)	94 (92–95)
Supplementary O_2_, lpm	2 (1–3)	3 (1–3)	2 (1–3)
Time from symptom onset to medical care, days	5 (3.5–5.0)	5 (3–5)	5 (4–5)
NEWS-2, score	6 (5–7)	5 (5–6)	7 (5–8)
COVID-19 severity, *n* (%)			
Mild	19 (18.1)	9 (17.6)	10 (18.5)
Moderate	86 (81.9)	42 (82.4)	44 (81.5)
Comorbidities			
Hypertension, *n* (%)	49 (46.7)	22 (43.1)	27 (50.0)
Ischemic heart disease, *n* (%)	5 (4.8)	4 (7.8)	1 (1.9)
Chronic kidney disease, *n* (%)	2 (1.9)	1 (2.0)	1 (1.9)
Heart failure, *n* (%)	1 (1.0)	1 (2.0)	0 (0.0)
Chronic liver disease, *n* (%)	1 (1.0)	1 (2.0)	0 (0.0)
Obstructive sleep apnea, *n* (%)	1 (1.0)	1 (2.0)	0 (0.0)
Diabetes, *n* (%)	43 (41.0)	20 (39.2)	23 (42.6)
Obesity, *n* (%)	46 (43.8)	23 (45.1)	23 (42.6)
Smoking, *n* (%)	21 (20.0)	12 (23.5)	9 (16.7)
Asthma, *n* (%)	3 (2.9)	2 (3.9)	1 (1.9)
COPD, *n* (%)	3 (2.9)	1 (2.0)	2 (3.7)
Dementia, *n* (%)	0 (0.0)	0 (0.0)	0 (0.0)
Rheumatologic disease, *n* (%)	1 (1.0)	0 (0.0)	1 (1.9)
HIV, *n* (%)	1 (1.0)	1 (2.0)	0 (0.0)
Cancer, *n* (%)	2 (1.9)	2 (3.9)	0 (0.0)
Immunosuppression, *n* (%)	1 (1.0)	1 (2.0)	0 (0.0)
Symptoms			
Myalgia, *n* (%)	55 (52.4)	28 (54.9)	27 (50.0)
Arthralgia, *n* (%)	47 (44.8)	24 (47.1)	23 (42.6)
Dyspnea, *n* (%)	92 (87.6)	45 (88.2)	47 (87.0)
Anosmia, *n* (%)	12 (11.4)	6 (11.8)	6 (11.1)
Fever, *n* (%)	66 (62.9)	29 (56.9)	37 (68.5)
Fatigue, *n* (%)	71 (67.6)	30 (58.8)	41 (75.9)
Headache, *n* (%)	46 (43.8)	26 (51.0)	20 (37.0)
Cough, *n* (%)	61 (58.1)	32 (62.7)	29 (53.7)
Diarrhea, *n* (%)	16 (15.2)	6 (11.8)	10 (18.5)
Nausea, *n* (%)	5 (4.8)	2 (3.9)	3 (5.6)
Vomiting, *n* (%)	2 (1.9)	1 (2.0)	1 (1.9)
Red eye, *n* (%)	2 (1.9)	1 (2.0)	1 (1.9)
Sore throat, *n* (%)	30 (28.6)	14 (27.5)	16 (29.6)
Chest pain, *n* (%)	24 (22.9)	10 (19.6)	14 (25.9)
Concomitant in-hospital medications			
Hydroxychloroquine, *n* (%)	0 (0.0)	0 (0.0)	0 (0.0)
Chloroquine, *n* (%)	0 (0.0)	0 (0.0)	0 (0.0)
Azithromycin, *n* (%)	10 (9.5)	4 (7.8)	6 (11.1)
Oseltamivir, *n* (%)	0 (0.0)	0 (0.0)	0 (0.0)
Statins, *n* (%)	8 (7.6)	6 (11.8)	2 (3.7)
Nitrates, *n* (%)	2 (1.9)	2 (3.9)	0 (0.0)
ACEI, *n* (%)	12 (11.4)	8 (15.7)	4 (7.4)
ARB, *n* (%)	28 (26.7)	11 (21.6)	17 (31.5)
Alteplase, *n* (%)	0 (0.0)	0 (0.0)	0 (0.0)
Ceftriaxone, *n* (%)	28 (26.7)	12 (23.5)	16 (29.6)
Ivermectin, *n* (%)	2 (1.9)	0 (0.0)	2 (3.7)
Nitazoxanide, *n* (%)	0 (0.0)	0 (0.0)	0 (0.0)
Metronidazole, *n* (%)	0 (0.0)	0 (0.0)	0 (0.0)
Aspirin, *n* (%)	9 (8.6)	4 (7.8)	5 (9.3)
Ibuprofen, *n* (%)	1 (1.0)	0 (0.0)	1 (1.9)
Indomethacin, *n* (%)	0 (0.0)	0 (0.0)	0 (0.0)
Systemic corticosteroid, *n* (%)	86 (81.9)	41 (80.4)	45 (83.3)
Colchicine, *n* (%)	0 (0.0)	0 (0.0)	0 (0.0)
Unfractionated heparin, *n* (%)	1 (1.0)	0 (0.0)	1 (1.9)
Low molecular weight heparin, *n* (%)	100 (95.2)	47 (92.2)	53 (98.1)
Convalescent plasma, *n* (%)	1 (1.0)	1 (2.0)	0 (0.0)
Remdesivir, *n* (%)	0 (0.0)	0 (0.0)	0 (0.0)
IV immunoglobulin, *n* (%)	0 (0.0)	0 (0.0)	0 (0.0)
Vitamin C, *n* (%)	7 (6.7)	5 (9.8)	2 (3.7)
Lopinavir-Ritonavir, *n* (%)	0 (0.0)	0 (0.0)	0 (0.0)

Quantitative data are presented as median and 1st-3rd quartile. Qualitative data are presented as the frequency with a percentage. *A full description of the calculation procedure of the PH-Covid19 risk score can be found in reference (31). ACEI, angiotensin-converting enzyme inhibitors; ARB, angiotensin-II receptor blockers; bpm, beats/breaths per minute; COPD, chronic obstructive pulmonary disease; COVID-19, coronavirus disease; DBP, diastolic blood pressure; HR, heart rate; HIV, human immunodeficiency virus; Kg, kilograms; lpm, liters per minute; m, meters; mmHg, millimeters of mercury; NEWS-2, National Early Warning Score 2; SpO_2_, peripheral arterial oxygen saturation; RR, respiratory rate; SBP, systolic blood pressure.

Thermotherapy was well tolerated since participants in the intervention group received a median of 10 (IQR: 8.75–10.0) thermotherapy sessions, 90 min each, with a median total duration of thermotherapy of 900 (IQR: 877.5–900) min.

**Figure 2:**
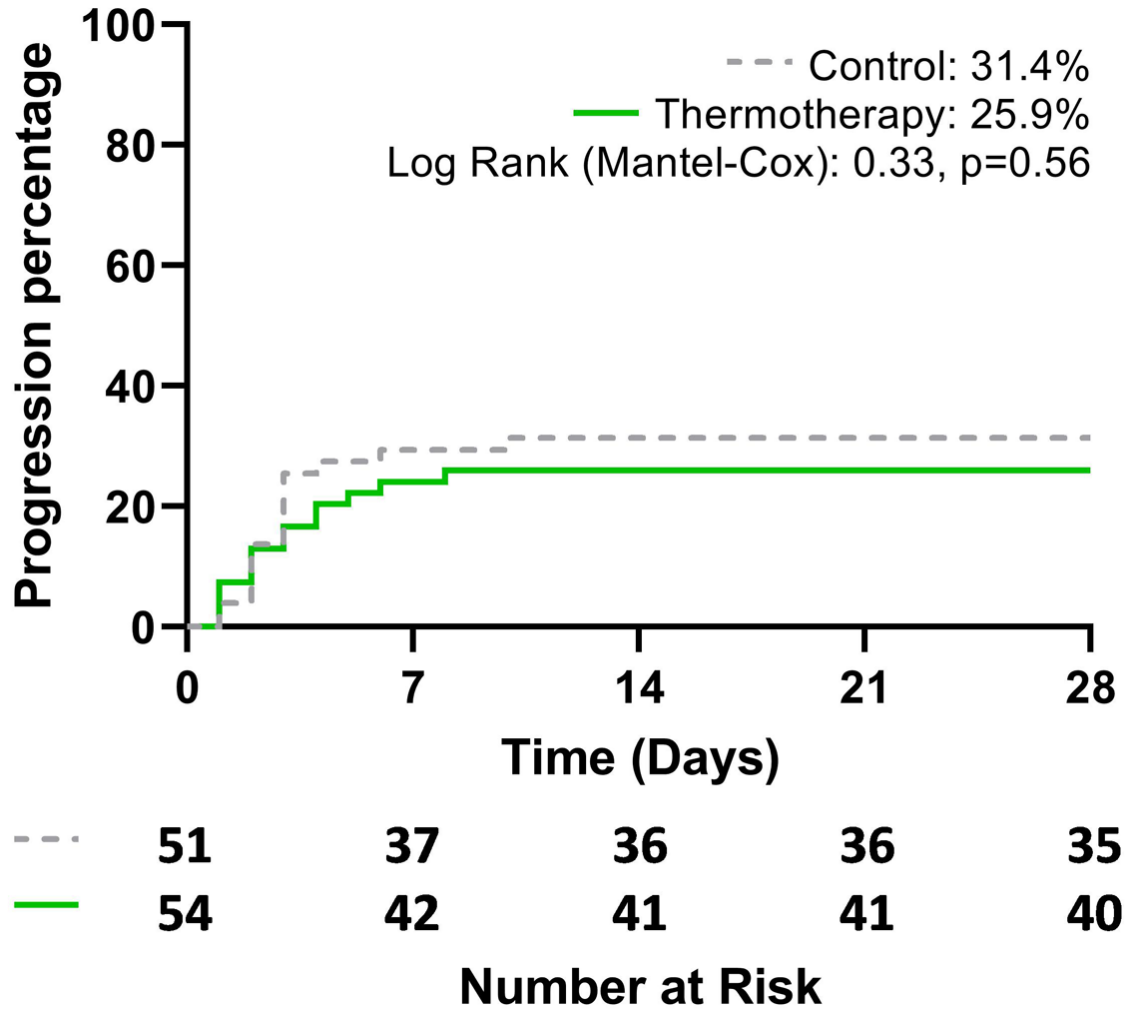
Cumulative probability of disease progression to severe-to-critical COVID-19 or death, according to the treatment group during the study period (28 days).

The primary outcome of disease progression occurred in 31.4% (16/51) of patients in the control group vs. 25.9% (14/54) of those who received the intervention, although this difference was not statistically significant (risk difference = 5.5%; 95% CI: −11.8–22.7, *p* = 0.54). Figure 2 shows the Kaplan–Meier plot for the cumulative probability of experiencing disease progression (*p* = 0.56). Most events of progression occurred during the first 7 days (control: 14/16, 87.5%; thermotherapy: 12/14, 85.7%). Progression to severe COVID-19 in the control group was 11.8% (6/51) and in the thermotherapy group it was 13.0% (7/54) (*p* = 0.85), while progression to critical COVID-19 was 19.6% (10/51) and 11.1% (6/54), respectively (*p* = 0.23). Time-to-progression of disease was not statistically significantly different among the control (7 days, IQR: 6.25–8.0) and thermotherapy (6.5 days, IQR: 5.75–9.25) groups (*p* = 0.78). Time-to-progression to critical COVID-19 was not statistically significantly different (11.0 days [IQR:9.25–14.0] vs. 13.0 days [9.75–15.0], *p* = 0.41). The comparisons in mortality, IMV, ICU admission, duration of hospitalization, VMI-free days, and time to admission to the ICU are shown in Table 3. There were no statistically significant differences in these secondary outcomes between the control and thermotherapy groups.

**Table 3:** Comparison of the secondary outcomes: mortality, hospitalization time, ICU admission, and IMV between study groups.

	Control	Thermotherapy	*p*-value
Mortality at day 15, *n*/total sample (%)	5/51 (62.5)	3/54 (37.5)	0.48
Mortality at day 28, *n*/total sample (%)	8/51 (57.1)	6/54 (42.9)	0.49
Hospitalization time, days	51/51 [6.0 (4.0–11.0)]	54/54 [7.0 (5.0–11.0)]	0.39
Requiring IMV, *n*/total sample (%)	6/51 (54.5)	5/54 (45.5)	0.67
Time to requiring IMV, days	6/51 [14 (10.75–18.25)]	6/51 [12.0 (9.50–14.50)]	0.31
Admission to ICU, *n*/total sample (%)	3/51 (42.9)	4/54 (57.1)	0.99
Time to requiring admission to ICU, days	3/51 [15.0 (11.0–15.0)]	4/54 [15.0 (11.0–18.25)]	0.86

Quantitative data are presented as median and 1st-3rd quartile. Qualitative data are presented as the frequency and percentage. The data are presented with the number of participants analyzed/number of participants in the study group. Quantitative comparisons were made using the Mann–Whitney U-test, and qualitative comparisons using the Chi-square test or Fisher’s exact test. ICU, intensive care unit; IMV, invasive mechanical ventilation.

**Figure 3:**
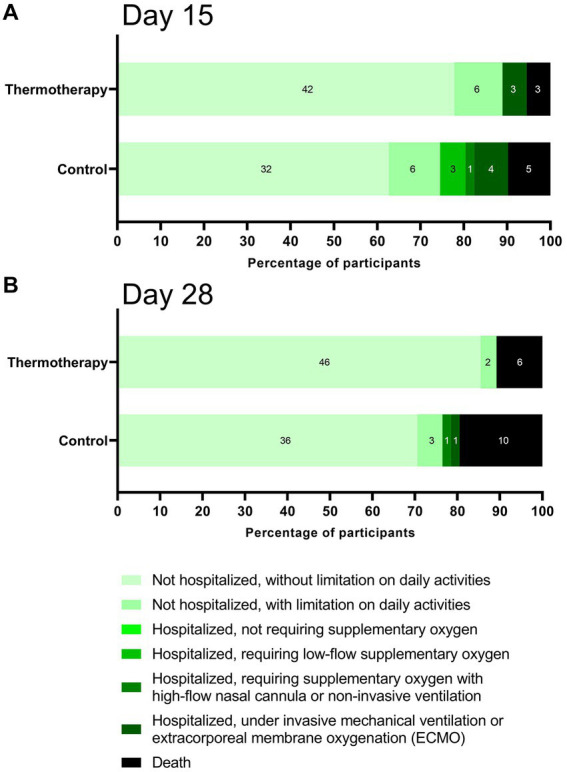
Percentage of participants at each clinical status on days **(A)** 15 and **(B)** 28.

Figure 3 shows the percentage of participants according to clinical status in the ordinal scale on days 15 (Figure 3A) and 28 (Figure 3B). Although the differences were not statistically significant (day 15: *p* = 0.10; day 28: p = 0.10), participants in the thermotherapy group had a trend toward a better recovery as shown by the higher proportion of patients not hospitalized and without a limitation on daily activities by days 15 (77.8% vs. 62.7%) and 28 (85.2% vs. 70.6%). By day 15, no patients in the thermotherapy group were in the hospitalized requiring low-flow or high-flow oxygen categories, which likely explained the higher proportion of recovered patients, similar to what was observed by day 28, with the addition of no patients under IMV in the thermotherapy group. Baseline laboratory values are presented in Supplementary Table S2.

**Figure 4:**
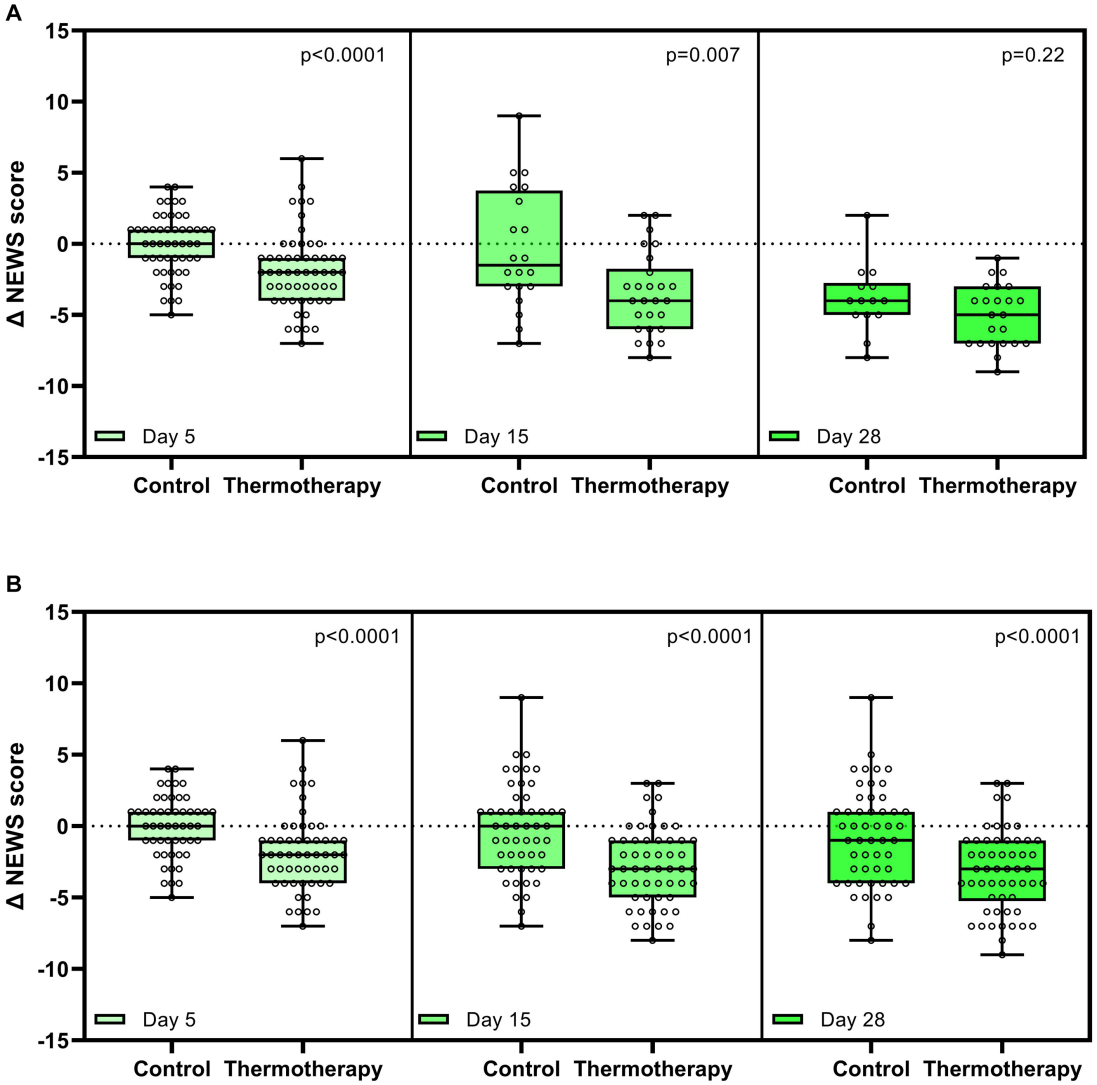
Change in National Early Warning Score 2 (NEWS-2) with respect to baseline on days 5, 15, and 28. **(A)** Comparisons between groups only in patients who were still hospitalized on days 5, 15, and 28. **(B)** Comparisons between groups considering the last observable data on days 5, 15, and 28. Data are presented as median, quartile 1, quartile 3 (Box), maximum, and minimum (whiskers). Dots represent the data of each patient. Comparison between the study groups was made by the Mann–Whitney U-test.

There was a greater improvement in clinical status according to the NEWS-2 score in patients treated with thermotherapy, as shown by the significant differences in the absolute change of the NEWS-2 score with respect to baseline by the end of the intervention (day 5) and day 15, but not at day 28 due to the low number of patients who remained hospitalized (Figure 4A). A non-prespecified analysis considering the latest NEWS-2 score of patients when still hospitalized was performed to elucidate what the potential difference could have been if patients had remained hospitalized at all time points (Figure 4B).

There were no significant differences in the total time that patients spent under different modalities of supplementary oxygen administration. For a simple nasal cannula or face mask, patients in the control group had a median time of 4.0 (IQR: 2.0–7.75) days, whereas patients in the thermotherapy group had a median of 5.0 (IQR: 3.0–7.75) days (*p* = 0.20). For the face mask with reservoir modality, 15 participants in the control group had it for a median of 3.0 (IQR: 1.0–7.0) days, and 13 patients in the thermotherapy group had it for 3.0 (IQR: 2.0–5.0) days (*p* = 0.83). Two patients in each group used high-flow nasal cannulas for a median of 15.5 (Q2 = 6.0) days in the control group and 6.0 (Q2 = 5.0) days in the thermotherapy group (*p* = 0.43). IMV was used in six patients in the control group for 3.0 (IQR: 0.0–11.0) days and five patients in the thermotherapy group for 6 (IQR: 5.50–12.0) days (*p* = 0.23).

**Figure 5:**
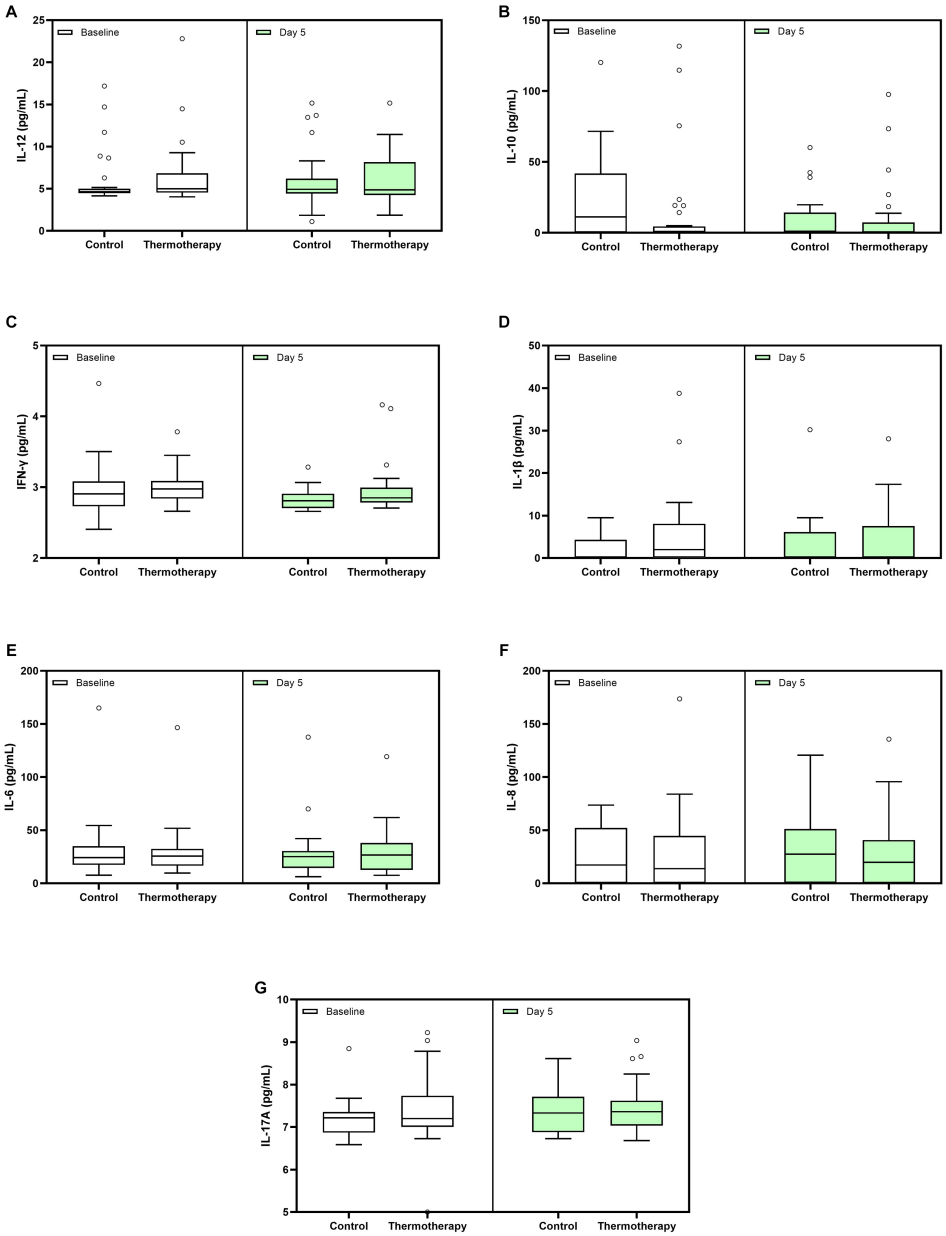
Whole blood cytokine levels at baseline and day 5 for both treatment groups. **(A)** IL-12, **(B)** IL-10, **(C)** IFN-γ, **(D)** IL-1β, **(E)** IL-6, **(F)** IL-8, and **(G)** IL-17A. Data are presented as median, quartile 1, quartile 3 (Box), and 1.5 times quartile 1 or 3 (whiskers). Dots represent outliers.

Absolute values of cytokine level determinations in the blood are shown in Figure 5. There were no significant differences among groups for the change in cytokine levels between day 5 and baseline (Table 4). The values of other inflammatory markers are shown in Supplementary Figure S1. There were no significant differences for any of the inflammatory markers (Supplementary Table S3) and other laboratory parameters (Supplementary Table S4) between the control and intervention groups.

**Table 4:** Comparison of cytokine levels at day 5 with respect to baseline between the study groups.

	Control	Thermotherapy	*p*-value
ΔIL-12, pg/mL	32/51 [0.10 (−0.39–0.56)]	34/54 [−0.27 (−1.46–0.98)]	0.37
Ratio of change^ǂ^	0.98	0.91	
ΔIL-10, pg/mL	32/51 [−5.30 (−26.44–0.00)]	34/54 [−0.11 (−2.86–0.09)]	0.10
Ratio of change^ǂ^	0.28	0.68	
ΔIFN-γ, pg/mL	32/51 [−0.02 (−0.24–0.03)]	33/54 [−0.02 (−0.14–0.01)]	0.78
Ratio of change^ǂ^	0.93	0.98	
ΔIL-1β, pg/mL	32/51 [0.00 (0.00–1.42)]	34/54 [0.00 (−4.49–0.35)]	0.15
Ratio of change^ǂ^	1.07	0.40	
ΔIL-6, pg/mL	32/51 [−3.12 (−8.95–5.40)]	34/54 [−0.68 (−6.35–6.14)]	0.41
Ratio of change^ǂ^	0.94	0.97	
ΔIL-8, pg/mL	32/51 [0.00 (−3.83–13.07)]	34/54 [0.00 (−13.50–10.50)]	0.28
Ratio of change^ǂ^	1.69	0.60	
ΔIL-17a, pg/mL	32/51 [0.04 (−0.18–0.41)]	33/54 [0.03 (−0.21–0.30)]	0.89
Ratio of change^ǂ^	1.01	0.99	

Cytokine levels are presented as median with 1st–3rd quartile. The data are presented as the number of participants analyzed/number of participants in the study group. Comparisons were made by the Mann–Whitney U-test. ǂ The ratio of change is presented as a geometric mean.

Seven (13.7%) patients in the control group and 7 (12.9%) in the thermotherapy group had at least one AE (*p* = 0.9). The absolute frequency of AEs by treatment group was 13 (control) and 16 (thermotherapy), with medians of 1.0 (IQR: 1.0–2.5) and 2.0 (IQR: 1.5–3.0) AEs in the control and thermotherapy groups, respectively. There were no significant differences in AE by outcome, severity, or causality (Supplementary Table S5). All AEs were classified as uncertain (likely explained by the natural history of disease, concomitant diseases, or drugs) in both treatment groups.

## Discussion

In this randomized controlled trial, we sought to study the efficacy and safety of mild thermotherapy locally administered through electronic heat pads in hospitalized patients with mild-to-moderate COVID-19 to prevent disease progression. We found that thermotherapy administered in the thorax continuously for 90 min, twice daily (every 12 h), for 5 days was well tolerated, and we did not observe any adverse effects with potential causal associations with the intervention. The proportion of patients receiving thermotherapy who experienced progression (25.9%) was lower compared to those receiving standard care (31.4%), but this difference was not statistically significant (*p* = 0.54). Patients in our study had higher progression rates than those in the report used for sample size estimation (13%) (21), which could be explained by the higher baseline risk in our study population due to comorbidities such as diabetes (41.0% vs. 11.0%), hypertension (46.7% vs. 22.7%), and smoking (20.0% vs. 16.3%), which are important risk factors for adverse outcomes in patients with COVID-19 (31). Obesity, which is one of the main factors for mortality in Mexican patients with COVID-19 (34), was also present in a high proportion of patients in our study (43.8% vs. not reported by Huang et al.).

Regarding the baseline prognosis of patients, the allocation method of minimization with a 20% random component was successful in equally distributing confounders closely related to adverse COVID-19 outcomes (sex, age, BMI, diabetes, hypertension, and supplementary oxygen at admission) and most other characteristics. Nonetheless, we observed differences in the baseline PH-COVID-19 risk score (31) with more patients in the thermotherapy group in the *high-risk* category (7.4% vs. 0%) and NEWS-2 score—higher in the thermotherapy group (median score: 7 vs. 5)—with certain symptoms reported at admission, some of which were higher in the thermotherapy (fever, fatigue, diarrhea, and chest pain) and others in control (headache and cough) groups. Such differences, despite having occurred due to chance, may imply a somewhat higher baseline risk for disease progression in the thermotherapy group. Regarding co-interventions that were being studied as potential treatments for COVID-19, these were well-balanced among groups, except for differences in the choice of anti-hypertensive medications and statins since patients in the control group tended to receive more ACEIs (15.7% vs. 7.4%) and statins (11.8% vs. 3.7%) and less ARBs (21.6% vs. 31.5%). These drugs do not affect COVID-19 prognosis according to systematic reviews of randomized controlled trials (35, 36).

Survival curves for disease progression show that the probability of progression was similar during the first 3 days, with a slightly higher probability of progression in the thermotherapy group in the first 2 days. This could be explained by the higher baseline risk in the thermotherapy group, as detailed earlier. After that, survival curves separated by day 4 onward, which could be attributable to the effect of the intervention, for which the latency period to observe any effects in the prevention of progression due to thermotherapy could be 48–72 h. Nonetheless, these differences could also be due to chance (wide and overlapping confidence intervals and non-significant differences in log-rank analysis). Since progression occurred during the first 7 days in most patients, future studies could use shorter time periods to assess progression (i.e., 7–14 days). In the pilot randomized controlled study by Bonfanti et al. (16), 19 participants with COVID-19 under invasive mechanical ventilation were randomized to receive an intervention consisting of increasing the core temperature with an esophageal core warming device to a target temperature of a maximum of 39.8°C or standard care. Although this study did not assess disease progressions as patients were included upon developing critical disease, no significant differences in mortality (22 and 30% in the intervention and control groups, respectively) were observed, although the study was not powered to assess differences in mortality.

Of note, the study was stopped early due to the dismantling of temporary COVID-19 medical units after decreasing infection rates in the country. Hence, the study was underpowered to detect differences in progression according to the sample size calculation (anticipated sample size of 274 patients vs. the final sample of 105). The hospital setting of this study made it difficult to recruit patients in the early stages of the disease (first 5 days) since these patients most commonly seek attention in ambulatory units. Although this was expected during the planning stage of the study, we opted for the hospital setting to be able to monitor tolerance and adherence to thermotherapy and establish safety under a more controlled in-hospital setting due to the limited knowledge of this new disease. Since thermotherapy was well-tolerated and safe, we believe that future studies aiming to test thermotherapy during the early stages of viral infections could be safely planned in ambulatory settings while also aiming to include women with confirmed or suspected pregnancy and other underrepresented patient populations (37). Bonfanti et al. (16) found that increasing the body core temperature was safe and feasible, although increasing the body core temperature was not achieved through a single device, which is why multimodal temperature management could improve thermal management. Thus, local thermotherapy could also be studied in the future in conjunction with core temperature management devices.

A similar picture was observed for most other secondary outcomes since no statistically significant differences were observed, possibly due to low statistical power. However, the clinical status on the ordinal scale shows that patients in the thermotherapy group tended to have a better recovery by days 15 and 28, as shown in Figure 3. Nonetheless, it is possible that some of the differences in clinical status could have been subject to bias since this was an open-label study and the WHO clinical progression scale is vulnerable to bias, especially at the lower end of the scale where assessment is most subjective (25). At the upper end of the scale, the tool is more vulnerable to confounding and preferred practices by physicians and centers.

Patients in the thermotherapy group had greater decreases in the NEWS-2 score by day 15 but not by day 28. Since this is a clinical evaluation carried out in-hospital, this analysis was especially affected by the loss of statistical power. For those reasons, we made a non-prespecified analysis by considering the last NEWS-2 score of patients in both treatment groups to get a picture of what could have happened had all patients been assessed by day 28. In such analysis, statistically significant differences implying a better recovery of patients who received thermotherapy remained, although these differences were only exploratory and should be interpreted with caution. It is also important to note that patients in the thermotherapy group had higher baseline NEWS-2 scores than the control group.

Regarding cytokine measurements, we hypothesized that thermotherapy would lead to a greater decline in pro-inflammatory cytokines. Unfortunately, we were not able to make 15- and 28-day measurements due to the insufficient number of patients still hospitalized to collect those samples. Thus, analyses were restricted to comparisons between day 5 (the last day of thermotherapy) and baseline. We did not observe differences between groups for any of the cytokines studied. While the prognostic value from baseline values in serum cytokine levels has been observed when comparing patients who developed severe-to-critical disease vs. those with mild COVID-19 (38), longitudinal trajectories are more complex. In the study by Sánchez-de Prada et al., no statistically significant differences were observed between survivors and non-survivors for IL-1β, IL-6, IL-8, IL-10, IL-12, IL-17, and IFN-γ at baseline and day 6 after hospital admission (39). In the studies by Liu et al. (40) and Han et al. (41), IL-6, IL-10, and IFN-γ were the only cytokines measured matching our study. In the first study, increased levels of these cytokines were reported in patients with severe COVID-19, compared to those with mild disease, 4–6 days after admission. Han et al. reported significant baseline differences in IL-6 and IL-10 but not IFN-γ when comparing patients with mild, moderate, and severe disease. By days 5–8, only differences in IL-6 persisted.

Limitations of our trial were that recruitment ended before reaching the target sample size, which limited our ability to more confidently interpret the findings; the open-label design due to the impossibility of blinding the intervention (a medical device providing heat) could have introduced bias, and the in-hospital setting made it difficult to recruit patients with mild-to-moderate disease presenting to hospital during the first days after symptom onset and importantly limited generalizability for the same reason. Future studies could attempt to assess thermotherapy in ambulatory settings to facilitate recruitment, although adherence to the intervention would be an additional challenge to consider in ambulatory settings.

This is the first randomized controlled trial that has evaluated local thermotherapy to prevent disease progression in patients with COVID-19. Another strength of our study was that thermotherapy was administered by supervised personnel, and we were able to establish the safety of the intervention. As found in a recent study, unifying clinical and epidemiological surveillance processes in Mexico is one of the main areas of opportunity to improve healthcare policies (42). Further research on low-cost interventions for treating infectious diseases could be an important piece to be incorporated into clinical practice and policymaking in future.

## Conclusion

Local thermotherapy administered in the thorax continuously for 90 min, twice daily (every 12 h), for 5 days in hospitalized patients with COVID-19 was well-tolerated and safe. A non-statistically significant lower proportion of patients who experienced disease progression was found in patients who received thermotherapy compared to those receiving standard care. Local thermotherapy could be further studied as a strategy to prevent disease progression in ambulatory settings.

## Data availability statement

The datasets presented in this study can be found in online repositories. The names of the repository/repositories and accession number(s) can be found below: Harvard Dataverse at https://doi.org/10.7910/DVN/QXBM3S.

## Ethics statement

The studies involving humans were approved by Dirección General de Calidad y Educación en Salud (CEI-DGCES/2020:03.1) and Instituto Nacional de Perinatología (2020-1-19). The studies were conducted in accordance with the local legislation and institutional requirements. The participants provided their written informed consent to participate in this study.

## Author contributions

JM-G: Conceptualization, Data curation, Formal analysis, Investigation, Methodology, Writing – original draft, Writing – review & editing. AK-G: Conceptualization, Formal analysis, Investigation, Methodology, Writing – original draft, Writing – review & editing, Data curation, Software, Visualization. MM-G: Data curation, Formal analysis, Investigation, Writing – original draft. JG: Project administration, Resources, Supervision, Writing – review & editing. YN: Data curation, Investigation, Supervision, Writing – review & editing. OS: Data curation, Investigation, Writing – review & editing. MT: Data curation, Investigation, Writing – review & editing. DM: Data curation, Investigation, Writing – review & editing. LF-U: Data curation, Investigation, Writing – review & editing. JR: Data curation, Investigation, Supervision, Writing – review & editing. IN: Data curation, Investigation, Writing – review & editing. AV-M: Data curation, Writing – review & editing. JM-R: Methodology, Supervision, Writing – review & editing. RF-D: Project administration, Writing – review & editing. NG-S: Conceptualization, Funding acquisition, Investigation, Methodology, Project administration, Resources, Supervision, Writing – original draft, Writing – review & editing.

## References

[1] Instituto Nacional de Estadística y Geografía. Estadísticas de Defunciones Registradas 2021. Mexico City; (2022).

[2] Instituto Nacional de Estadística y Geografía. Estadísticas de Defunciones Registradas 2022 (Preliminar). Mexico City; (2023).

[3] Basto-Abreu A Carnalla M Torres-Ibarra L Sanchez-Pájaro A Romero-Martínez M Martínez-Barnetche J. SARS-CoV-2 seroprevalence and vaccine coverage from august to November 2021: a nationally representative survey in Mexico. J Med Virol. (2023) 95:e29038. doi: 10.1002/jmv.29038, PMID: 37615363

[4] Smith J. Latin America roundup: COFEPRIS ends emergency fast tracking for new COVID vaccines. Regulatory Focus: Regulatory Affairs Professionals Society. (2023)

[5] Hampton A-R Eccleston-Turner M Rourke M Switzer S. Equity in the pandemic treaty: access and benefit-sharing as a policy device or a rhetorical device? J Law Med Ethics. (2023) 51:217–20. doi: 10.1017/jme.2023.59, PMID: 37226758 PMC10209987

[6] Flahault A Calmy A Costagliola D Drapkina O Eckerle I Larson HJ. No time for complacency on COVID-19 in Europe. Lancet. (2023) 401:1909–12. doi: 10.1016/S0140-6736(23)01012-7 37230103 PMC10202416

[7] Huang W Hernandez I Tang S Dickson S Berenbrok LA Guo J. Association between distance to community health care facilities and COVID-19–related mortality across U.S. counties in the COVID-19–vaccine era. BMC Res Notes. (2023) 16:96. doi: 10.1186/s13104-023-06366-3 37277859 PMC10241387

[8] Lamontagne F Agarwal A Rochwerg B Siemieniuk RA Agoritsas T Askie L. A living WHO guideline on drugs for covid-19. BMJ. (2020) 370:m3379. doi: 10.1136/bmj.m3379 32887691

[9] Plata GG. The black market for covid-19 antiviral drugs. BMJ. (2022) 377:o1282. doi: 10.1136/bmj.o1282 35640944

[10] Mancilla-Galindo J Galindo-Sevilla N. Exploring the rationale for thermotherapy in COVID-19. Int J Hyperth. (2021) 38:202–12. doi: 10.1080/02656736.2021.1883127, PMID: 33682604

[11] Gazel D Yılmaz M. Are infectious diseases and microbiology new fields for thermal therapy research? Int J Hyperth. (2018) 34:918–24. doi: 10.1080/02656736.2018.1440015, PMID: 29448846

[12] Cohen M. Turning up the heat on COVID-19: heat as a therapeutic intervention. F1000Research. (2020) 9:292. doi: 10.12688/f1000research.23299.1 32742639 PMC7372531

[13] Ramirez FE Sanchez A Pirskanen AT. Hydrothermotherapy in prevention and treatment of mild to moderate cases of COVID-19. Med Hypotheses. (2021) 146:110363. doi: 10.1016/j.mehy.2020.110363, PMID: 33303302 PMC7668174

[14] Liikkanen LA Laukkanen JA. Sauna bathing frequency in Finland and the impact of COVID-19. Complement Ther Med. (2020) 2021:102594. doi: 10.1016/j.ctim.2020.102594 33197669

[15] Masaud SM Szasz O Szasz AM Ejaz H Anwar RA Szasz A. A potential bioelectromagnetic method to slow down the progression and prevent the development of ultimate pulmonary fibrosis by COVID-19. Front Immunol. (2020) 11:1–14. doi: 10.3389/fimmu.2020.556335 33343561 PMC7746880

[16] Bonfanti NP Mohr NM Willms DC Bedimo RJ Gundert E Goff KL. Core warming of coronavirus disease 2019 patients undergoing mechanical ventilation: a pilot study. Ther Hypothermia Temp Manag. (2023) 13:225–9. doi: 10.1089/ther.2023.0030 37527424 PMC10698775

[17] Tian F Wang J Xi X Sun X He M Zhao C. Efficacy and safety of short-wave diathermy treatment for moderate COVID-19 patients: a prospective, double-blind, randomized controlled clinical study. Eur J Phys Rehabil Med. (2022) 58:137–43. doi: 10.23736/S1973-9087.21.06892-1, PMID: 34042412 PMC9980486

[18] Huang L Li Q Shah SZA Nasb M Ali I Chen B. Efficacy and safety of ultra-short wave diathermy on COVID-19 pneumonia: a pioneering study. Front Med. (2023) 10:1149250. doi: 10.3389/fmed.2023.1149250 PMC10277738 37342496

[19] Wu Z McGoogan JM. Characteristics of and important lessons from the coronavirus disease 2019 (COVID-19) outbreak in China. JAMA. (2020) 323:1239–42. doi: 10.1001/jama.2020.2648, PMID: 32091533

[20] Gandhi RT Lynch JB del Rio C. Mild or moderate Covid-19. N Engl J Med. (2020) 383:1757–66. doi: 10.1056/NEJMcp2009249 32329974

[21] Huang J Cheng A Lin S Zhu Y Chen G. Individualized prediction nomograms for disease progression in mild COVID-19. J Med Virol. (2020) 92:2074–80. doi: 10.1002/jmv.25969, PMID: 32369205 PMC7267495

[22] American Society of Health-System Pharmacists. (2020). Assessment of Evidence for COVID-19-Related Treatments: Updated 6/18/2020 [Internet]. American Society of Health-System Pharmacists (ASHP) COVID-19 Resources, p. 63. Available at: https://www.ashp.org/-/media/assets/pharmacy-practice/resource-centers/Coronavirus/docs/ASHP-COVID-19-Evidence-Table.ashx

[23] Wang Y Fan G Horby P Hayden F Li Q Wu Q. Comparative outcomes of adults hospitalized with seasonal influenza a or B virus infection: application of the 7-category ordinal scale. Open forum. Infect Dis. (2019) 6:ofz053. doi: 10.1093/ofid/ofz053 PMC6419989 30895200

[24] Royal College of Physicians. National Early Warning Score (NEWS) 2: Standardising the assessment of acute-illness severity in the NHS, vol. 17. Updated report of a working party. 1st ed. London: RCP (2017).

[25] Marshall JC Murthy S Diaz J Adhikari NK Angus DC Arabi YM. A minimal common outcome measure set for COVID-19 clinical research. Lancet Infect Dis. (2020) 20:e192–7. doi: 10.1016/S1473-3099(20)30483-7, PMID: 32539990 PMC7292605

[26] Mexican Secretariat of Health. Norma Oficial Mexicana NOM-220-SSA1-2012, Instalación y operación de la farmacovigilancia [Internet]. Ciudad de México; (2012). p. 24. Available at: https://dof.gob.mx/nota_detalle_popup.php?codigo=5284236

[27] U.S. Department of Health and Human Services. Common Terminology Criteria for Adverse Events (CTCAE).v.5.0 [Internet]. Cancer Therapy Evaluation Program (CTEP). (2017). p. 155. Available at: https://ctep.cancer.gov/protocolDevelopment/electronic_applications/ctc.htm#ctc_50

[28] Guyatt GH Briel M Glasziou P Bassler D Montori VM. Problems of stopping trials early. BMJ. (2012) 344:e3863–3. doi: 10.1136/bmj.e3863 22705814

[29] O’Callaghan CA. OxMaR: open source free software for online minimization and randomization for clinical trials. PLoS ONE. (2014) 9:e110761. doi: 10.1371/journal.pone.0110761 25353169 PMC4213009

[30] Guillaumes S O’Callaghan CA. Versión en español del software gratuito OxMaR para minimización y aleatorización de estudios clínicos. Gac Sanit. (2019) 33:395–7. doi: 10.1016/j.gaceta.2018.07.013, PMID: 30390995

[31] Mancilla-Galindo J Vera-Zertuche JM Navarro-Cruz AR Segura-Badilla O Reyes-Velázquez G Tepepa-López FJ. Development and validation of the patient history COVID-19 (PH-Covid19) scoring system: a multivariable prediction model of death in Mexican patients with COVID-19. Epidemiol Infect. (2020) 148:e286. doi: 10.1017/S0950268820002903, PMID: 33239114 PMC7729170

[32] Dayim A. Consort: Create consort diagram [internet]. (2023). Available at: https://cran.r-project.org/package=consort

[33] R Core Team. R: A language and environment for statistical computing [internet]. Viena, Austria: R Foundation for Statistical Computing (2023).

[34] Vera-Zertuche JM Mancilla-Galindo J Tlalpa-Prisco M Aguilar-Alonso P Aguirre-García MM Segura-Badilla O. Obesity is a strong risk factor for short-term mortality and adverse outcomes in Mexican patients with COVID-19: a national observational study. Epidemiol Infect. (2021) 149:e109. doi: 10.1017/S0950268821001023, PMID: 33913410 PMC8134888

[35] Ren Y Wang G Han D. Statins in hospitalized COVID-19 patients: a systematic review and meta-analysis of randomized controlled trials. J Med Virol. (2023) 95:e28823. doi: 10.1002/jmv.28823, PMID: 37254831

[36] Asiimwe IG Pushpakom SP Turner RM Kolamunnage-Dona R Jorgensen AL Pirmohamed M. Cardiovascular drugs and COVID-19 clinical outcomes: a systematic review and meta-analysis of randomized controlled trials. Br J Clin Pharmacol. (2022) 88:3577–99. doi: 10.1111/bcp.15331, PMID: 35322889 PMC9111446

[37] Bierer BE Meloney LG Ahmed HR White SA. Advancing the inclusion of underrepresented women in clinical research. Cell Rep Med. (2022) 3:100553. doi: 10.1016/j.xcrm.2022.100553, PMID: 35492242 PMC9043984

[38] Zhang H Wu H Pan D Shen W. D-dimer levels and characteristics of lymphocyte subsets, cytokine profiles in peripheral blood of patients with severe COVID-19: a systematic review and meta-analysis. Front Med. (2022) 9:988666. doi: 10.3389/fmed.2022.988666, PMID: 36275800 PMC9579342

[39] Sánchez-de Prada L Gorgojo-Galindo Ó Fierro I Martínez-García AM de Quintana GS-L Gutiérrez-Bustillo R. Time evolution of cytokine profiles associated with mortality in COVID-19 hospitalized patients. Front Immunol. (2022) 13:1–10. doi: 10.3389/fimmu.2022.946730 PMC9551198 36238287

[40] Liu J Li S Liu J Liang B Wang X Wang H. Longitudinal characteristics of lymphocyte responses and cytokine profiles in the peripheral blood of SARS-CoV-2 infected patients. EBioMedicine. (2020) 55:102763. doi: 10.1016/j.ebiom.2020.102763, PMID: 32361250 PMC7165294

[41] Han H Ma Q Li C Liu R Zhao L Wang W. Profiling serum cytokines in COVID-19 patients reveals IL-6 and IL-10 are disease severity predictors. Emerg Microbes Infect. (2020) 9:1123–30. doi: 10.1080/22221751.2020.1770129, PMID: 32475230 PMC7473317

[42] Bautista-Reyes D Werner-Sunderland J Aragón-Gama AC Duran JRC Medina KDC Urbina-Fuentes M. Health-care policies during the COVID-19 pandemic in Mexico: a continuous case of heterogeneous, reactive, and unequal response. Health Policy Open. (2023) 5:100100. doi: 10.1016/j.hpopen.2023.100100, PMID: 37662095 PMC10471918

[43] Perez-Padilla R Torre-Bouscoulet L Muiño A Marquez MN Lopez MV de Oca MM. Prevalance of oxygen desaturation and use of oxygen at home in adults at sea level and at moderate altitude. Eur Respir J. (2006) 27:594–9. doi: 10.1183/09031936.06.00075005, PMID: 16507861

[44] Vargas-Domínguez C Gochicoa-Rangel L Velázquez-Uncal M Mejía-Alfaro R Vázquez-García JC Pérez-Padilla R. Pruebas de función respiratoria, ¿cuál y a quién? Neumol Cir Torax. (2011) 70:101–17.

[45] Rivera Pérez R David Suárez Nadal E Zajarias Kurschansky A De Las Deses De Juillac Wiechers H. Saturación de oxígeno en adultos mayores de la Ciudad de México. An Méd. (2008) 53:5–9.

