# Supplementary material for: Regional moderate hyperthermia for mild-to-moderate COVID-19 (TherMoCoV study): a randomized controlled trial
Source: Front Med (Lausanne). 2023 Dec 22;10:1256197. doi: 10.3389/fmed.2023.1256197 (PMC10766786; doi:10.3389/fmed.2023.1256197)
Supplement: Supplementary file 1 [file Data_Sheet_1.docx]

**Supplementary Table S1.** Characteristics of cytokine assays and reference units by cytokine.

| **Cytokine** | **Kit** | **Catalog** | **Type** | **Units** | **Reference values** |
| --- | --- | --- | --- | --- | --- |
| **IL-1β** | Invitrogen_(Viena, Austria)_ | BMS224-2 | Quantitative | pg/mL | 0.5-12 pg/mL^1^ |
| **IL-6** | ENZO | Enz-kit178-0001 | Quantitative | pg/mL | 0.5-43.5 pg/mL^2^ |
| **IL-8** | Invitrogen _(USA)_ | KAC1301 | Quantitative | pg/mL | 24.4-35.9 pg/mL^3^ |
| **IL-10** | Invitrogen _(USA)_ | KHC01101 | Quantitative | pg/mL | 8.5-16.7 pg/mL ^4^ |
| **IL-12 (p70)** | Invitrogen _(USA)_ | KAC1568 | Quantitative | pg/mL | 19.6-56.3 pg/mL^3^ |
| **IL-17A** | ENZO | ADI-900-177 | Quantitative | pg/mL | 16.07-40.77 pg/mL^5^ |
| **IFN-γ** | Invitrogen _(USA)_ | BMS228 | Quantitative | pg/mL | 1-132 pg/mL^6^ |

1. O’Neill CM, Lu C, Corbin KL, et al. Circulating Levels of IL-1B+IL-6 Cause ER Stress and Dysfunction in Islets From Prediabetic Male Mice. Endocrinology. 2013;154(9):3077. doi:10.1210/EN.2012-2138

2. Said EA, Al-Reesi I, Al-Shizawi N, et al. Defining IL-6 levels in healthy individuals: A meta-analysis. J Med Virol. 2021;93(6):3915-3924. doi:10.1002/JMV.26654

3. Kleiner G, Marcuzzi A, Zanin V, Monasta L, Zauli G. Cytokine levels in the serum of healthy subjects. Mediators Inflamm. 2013;2013. doi:10.1155/2013/434010

4. Gasim S, Elhassan AM, Khalil EAG, et al. High levels of plasma IL-10 and expression of IL-10 by keratinocytes during visceral leishmaniasis predict subsequent development of post-kala-azar dermal leishmaniasis. Clin Exp Immunol. 1998;111(1):64. doi:10.1046/J.1365-2249.1998.00468.X

5. Jiang S, Shan F, Zhang Y, Jiang L, Cheng Z. Increased serum IL-17 and decreased serum IL-10 and IL-35 levels correlate with the progression of COPD. Int J Chron Obstruct Pulmon Dis. 2018;13:2483-2494. doi:10.2147/COPD.S167192

6. Contoli M, Papi A, Tomassetti L, et al. Blood Interferon-α Levels and Severity, Outcomes, and Inflammatory Profiles in Hospitalized COVID-19 Patients. Front Immunol. 2021;12:536. doi:10.3389/FIMMU.2021.648004

**Supplementary Table S2**. Baseline laboratory values per treatment group.

|  | **Total** | | **Control** | | **Thermotherapy** | |
| --- | --- | --- | --- | --- | --- | --- |
| **Laboratory Parameter, units** | **Number analized/Total sample** | **Median (IQR)** | **Number analized/Total sample** | **Median (IQR)** | **Number analized/Total sample** | **Median (IQR)** |
| Glucose, mg/dL | 103/105 | 132.00 (105.00-184.00) | 51/51 | 129.00 (100.00-222.00) | 52/54 | 140.00 (107.25-182.75) |
| Urea, mg/dL | 103/105 | 32.10 (24.00-43.70) | 51/51 | 34.00 (23.00-43.70) | 52/54 | 31.00 (24.00-43.75) |
| Blood urea nitrogen, mg/dL | 103/105 | 15.87 (11.20-20.53) | 51/51 | 15.88 (10.74-20.09) | 52/54 | 14.75 (11.21-20.91) |
| Creatinine, mg/dL | 103/105 | 0.70 (0.60-0.90) | 51/51 | 0.70 (0.60-0.80) | 52/54 | 0.70(0.60-0.98) |
| Total bilirubin, mg/dL | 86/105 | 0.50 (0.40-0.70) | 39/51 | 0.50 (0.40-0.80) | 47/54 | 0.50 (0.40-0.70) |
| Direct bilirubin, mg/dL | 86/105 | 0.20 (0.10-0.30) | 39/51 | 0.20 (0.10-0.30) | 47/54 | 0.20 (0.10-0.30) |
| Indirect bilirubin, mg/dL | 86/105 | 0.30 (0.20-0.50) | 39/51 | 0.30 (0.20-0.60) | 47/54 | 0.30 (0.20-0.50) |
| AST, IU/L | 93/105 | 43.00 (31.00-55.50) | 44/51 | 40.50 (30.25-55.75) | 49/54 | 44.00 (31.50-55.50) |
| ALT, IU/L | 93/105 | 37.00 (23.00-61.50) | 44/51 | 37.00 (23.00-61.00) | 49/54 | 35.00 (24.00-63.00) |
| CPK, IU/L | 45/105 | 71.00 (36.00-146.00) | 23/51 | 66.00 (32.00-116.00) | 22/54 | 74.50 (37.00-153.50) |
| Hemoglobin, g/dL | 103/105 | 15.00 (13.50-16.00) | 51/51 | 15.40 (13.80-16.60) | 52/54 | 14.55 (13.43-15.70) |
| Hematocrit, % | 104/105 | 44.70 (41.23-48.30) | 51/51 | 46.10 (41.70-49.50) | 53/54 | 43.60 (40.80-47.55) |
| Monocytes, 10^3^/mL | 104/105 | 0.31 (0.21-0.47) | 51/51 | 0.33 (0.21-0.60) | 53/54 | 0.30 (0.21-0.42) |
| Platelets, 10^3^/mL | 104/105 | 221.50 (178.25-293.75) | 51/51 | 222.00 (171.00-301.00) | 53/54 | 221.00 (181.50-293.50) |
| Neutrophil-to-lymphocyte ratio | 104/105 | 4.85 (2.88-9.57) | 51/51 | 5.04(2.85-10.11) | 53/54 | 4.72 (2.90-9.08) |
| INR | 93/105 | 1.05 (0.99-1.12) | 46/51 | 1.06 (1.00-1.15) | 47/54 | 1.05 (0.98-1.11) |
| Prothrombin time, seconds | 93/105 | 14.30 (13.65-15.40) | 46/51 | 14.30(13.70-15.73) | 47/54 | 14.50 (13.50-15.30) |
| Partial thromboplastin time, seconds | 93/105 | 34.30 (30.65-38.20) | 46/51 | 34.85 (30.63-38.95) | 47/54 | 33.90 (30.60-37.90) |

Abbreviations: aspartate aminotransferase (AST), alanine aminotransferase (ALT), creatinine phosphokinase (CPK), international normalized ratio (INR)

**Suppl. Table S3.** Comparison of other inflammatory markers at day 5 and day 15 with respect to baseline.

|  | **Day 5** | | | **Day 15** | | |
| --- | --- | --- | --- | --- | --- | --- |
| **Laboratory parameter, units** | **Control** | **Thermotherapy** | **p value** | **Control** | **Thermotherapy** | **p value** |
| Total leucocytes, 10^3^/mL | 43/51 [1.20 (-2.20-3.70)] | 50/54 [1.59 (-0.30-3.35)] | 0.33 | 8/51 [4.20 (-4.25-12.82)] | 5/54 [0.10 (-0.75-7.40)] | 0.77 |
| Ratio of change^ǂ^ | 1.09 | 1.21 |  | 1.44 | 1.28 |  |
| Neutrophils, 10^3^/mL | 44/51 [0.32 (-2.43-329)] | 50/54 [1.09 (-0.88-2.58)] | 0.50 | 8/51 [3.76 (-4.03-12.3)] | 5/54 [-0.84 (-1.17-6.58)] | 0.88 |
| Ratio of change^ǂ^ | 1.07 | 1.18 |  | 1.36 | 1.16 |  |
| Lymphocytes, 10^3^/mL | 44/51 [-0.04 (-0.31-0.43)] | 50/54 [0.11 (-0.23-0.92)] | 0.09 | 8/51 [0.08 (-0.02-0.38)] | 5/54 [0.50 (-0.58-0.96)] | 0.66 |
| Ratio of change^ǂ^ | 1.05 | 1.17 |  | 1.28 | 1.34 |  |
| Albumin, g/dL | 41/51 [0.00 (-0.65-0.20)] | 43/54 [-0.30 (-0.60-0.00)] | 0.17 | 8/51 [-1.70 (-2.05- -0.42)] | 4/54 [-0.85 (-1.40-0.30)] | 0.24 |
| Ratio of change^ǂ^ | 0.94 | 0.91 |  | 0.64 | 0.82 |  |
| C-reactive protein (CRP), mg/L | 38/51 [-14.25 (-104.1-1.19)] | 45/54 [-28.80 (-115.8-10.7)] | 0.92 | 4/51 [15.2 (-247.5-57.74)] | 2/54 [-162 (-237.3- -161)] | 0.35 |
| Ratio of change^ǂ^ | 0.39 | 0.37 |  | 0.79 | 0.09 |  |
| Procalcitonin, ng/mL | 36/51 [0.00 (-0.12-0.06)] | 47/54 [-0.02 (-0.14-0.04)] | 0.78 | 4/51 [0.06 (-0.07-0.40)] | 3/54 [-0.04 (-0.17- -0.04)] | 0.72 |
| Ratio of change^ǂ^ | 0.91 | 0.71 |  | 1.13 | 2.02 |  |
| Lactate dehydrogenase (LDH), IU/mL | 42/51 [0.00 (-51.00-63.75)] | 45/54 [-9.00 (-60.00-77.50)] | 0.46 | 6/51 [172.5 (-74.7-290.5)] | 4/54 [-107 (-186.5-243.2)] | 0.52 |
| Ratio of change^ǂ^ | 1.07 | 1.05 |  | 1.26 | 0.92 |  |
| D-dimer, ng/mL | 38/51 [90.52 (-81.46-557.0)] | 44/54 [0.00 (-367.8-256.4)] | 0.07 | 4/51 [1445 (7.91-12556)] | 5/54 [756 (260-819)] | 1.00 |
| Ratio of change^ǂ^ | 1.36 | 0.98 |  | 0.99 | 3.18 |  |
| Fibrinogen, mg/dL | 6/51 [0.00 (-46.52-39.00)] | 5/54 [126.0 (56.0-163.5)] | 0.08 | No data | No data | 0.38 |
| Ratio of change^ǂ^ | 0.99 | 1.35 |  |  |  |  |
| Erythrocyte sedimentation rate (ESR), mm/h | 36/51 [0.00 (-4.80-2.75)] | 44/54 [0.00 (-4.00-1.50)] | 0.94 | 2/51 [-2.00 (-10.00- -2.00)] | 2/54 [-12.45 (-12.9- -12.45 )] | 0.12 |
| Ratio of change^ǂ^ | 0.88 | 0.89 |  | 0.67 | 0.06 |  |

Inflammatory markers level is presented as median with 1st-3rd quartile. The data are presented with the number of participants analyzed/number of participants in the study group.

The ratio of change is presented as geometric mean. Comparisons were made by Mann-Whitney U test.

**Supplementary Table S4**. Comparison of other laboratory parameters at day 5 and day 15 with respect to baseline.

|  | **Day 5** | | | **Day 15** | | |
| --- | --- | --- | --- | --- | --- | --- |
| **Laboratory parameter, units** | **Control**  **n=51** | **Thermotherapy**  **n=54** | **p value** | **Control**  **n=51** | **Thermotherapy**  **n=54** | **p value** |
| Glucose, mg/dL | 43/51 [-1.0 (-40.0-33.0)] | 49/54 [-3.0 (-24.5-11.5)] | 0.71 | -1.00 (-61.00-35.00) | -70 (-93.50-16.50) | 0.09 |
| Urea, mg/dL | 43/51 [5.00 (-4.00-12.80)] | 49/54 [2.00 (-4.50-13.00)] | 0.45 | 12.00 (-6.00-24.00) | 1.34 (-13.00-112.00) | 0.68 |
| Blood urea nitrogen, mg/dL | 43/51 [2.34 (-1.87-6.00)] | 49/54 [0.43 (-2.10-6.06)] | 0.48 | 5.60 (-2.81-11.21) | 0.62 (-5.90-52.34) | 0.68 |
| Creatinine, mg/dL | 43/51 [0.00 (-0.10-0.10)] | 49/54 [0.00 (-0.15-0.05)] | 0.75 | 0.00 (-0.20-0.30) | 0.10 (-0.30-0.45) | 1.00 |
| Total bilirubin, mg/dL | 41/51 [0.00 (-0.10-0.05)] | 43/54 [0.00 (-0.10-0.20)] | 0.23 | 0.00 (-0.32-0.27) | 0.15 (-0.15-1.2) | 0.52 |
| Direct bilirubin, mg/dL | 41/51 [0.00 (-0.10-0.10)] | 43/54 [0.00 (-0.10-0.00)] | 0.49 | 0.00 (-0.12-0.25) | 0.05 (0.00-0.40) | 0.44 |
| Indirect bilirubin, mg/dL | 41/51 [0.00 (-0.10-0.10)] | 43/54 [0.00 (0.00-0.10)] | 0.24 | 0.00 (-0.37-0.15) | 0.15 (-0.22-0.82) | 0.45 |
| AST, IU/L | 0.00 (-10.50-12.50) | 1.00 (-12.75-17.00) | 0.67 | 2.50 (-16.25-16.5) | -8.50 (-121.0-166.25) | 0.83 |
| ALT, IU/L | 5.00 (-4.00-24.00) | 2.00 (-1.00-35.00) | 0.59 | 9.00 (1.50-113.2) | 55.0 (-112.5-579.5) | 0.83 |
| CPK, IU/L | 0.00 (-30.00-0.00) | 0.00 (-35.50-0.00) | 0.67 | -177.0 (-354.0- -177.0) | No data | 0.99 |
| Hemoglobin, g/dL | 43/51 [0.00 (-1.00-1.00)] | 50/54[0.00 (-0.25-1.00)] | 0.14 | -2.95 (-3.22- -1.92) | -0.70 (-3.20- -0.35) | 0.46 |
| Hematocrit, % | -1.50 (-3.50-1.80) | -0.50 (-2.92-1.77) | 0.46 | -6.00 (-9.50- -4.52) | -2.60 (-10.90-0.00) | 0.56 |
| Monocytes, 10^3^/mL | 50/51 [0.01 (-0.14-0.19)] | 53/54 [0.14 (-0.04-0.38)] | 0.04 | 0.06 (0.01-0.31) | 0.26 (0.13-0.36) | 0.19 |
| Platelets, 10^3^/mL | 43/51 [94.0 (39.0-137.0)] | 49/54 [81.0 (20.5-158.5)] | 0.61 | 164.5 (71.25-211.25) | 235 (-11.0-320.5) | 0.46 |
| Neutrophil-to-lymphocyte ratio | 0.06 (-2.58-3.90) | 0.30 (-3.93-3.26) | 0.54 | 4.39 (-10.22-17.69) | -2.40 (-8.89-15.07) | 0.77 |
| INR | 0.00 (-0.05-0.05) | 0.02 (0.00-0.06) | 0.11 | 0.01 (0.00-0.31) | 0.09 (0.00-0.09) | 0.86 |
| Prothrombin time, seconds | 0.00 (0.95-0.77) | 0.30 (0.00-0.87) | 0.14 | 0.15 (0.03-4.70) | 1.50 (0.00-1.50) | 0.86 |
| Partial thromboplastin time, seconds | -2.45 (-7.77-0.00) | -1.20 (-5.62-0.37) | 0.26 | 0.30 (-6.67-2.17) | 0.00 (-4.70-0.00) | 0.72 |

Laboratory parameters presented as median with 1st-3rd quartile. The data are presented with the number of participants analyzed/number of participants in the study group.

The ratio of change is presented as geometric mean. Comparisons were made by Mann-Whitney U test.

Abbreviations: aspartate aminotransferase (AST), alanine aminotransferase (ALT), creatinine phosphokinase (CPK), international normalized ratio (INR).

**Supplementary Table S5.** Adverse events according to treatment group.

|  | Control group | Thermotherapy group | p value |
| --- | --- | --- | --- |
| Any adverse event, n (%) | 7/51 (13.7) | 7/54 (12.9) | 0.90 |
| Absolute frequency of adverse events per group | 13 | 16 | - |
| Adverse events per patient, median (IQR) | 1.0 (1.0, 2.5) | 2.0 (1.5, 3.0) | - |
| Proportion of adverse events according to outcome, n (%) | | | |
| Non-severe | 5 (38.5) | 7 (43.8) | 0.77 |
| Severe | 8 (61.5) | 9 (56.4) |  |
| Proportion of adverse events according to severity, n (%) | | | |
| Mild (Grade 1) | 6 (46.2) | 6 (37.5) | 0.41 |
| Moderate (Grade 2) | 0 (0.0) | 2 (12.5) |  |
| Severe (Grades 3, 4, and 5) | 7 (53.8) | 8 (50.0) |  |
| Proportion of adverse events according to causality, n (%) | | | |
| Certain | 0 (0.0) | 0 (0.0) | - |
| Probable | 0 (0.0) | 0 (0.0) |  |
| Possible | 0 (0.0) | 0 (0.0) |  |
| Uncertain | 13 (100.0) | 16 (100.0) |  |
| Unclassifiable | 0 (0.0) | 0 (0.0) |  |

Data presented as frequency with percentage of median with 1st-3rd quartile.

Comparisons were made with the chi-squared test.

**Supplementary Figure S1.** Whole blood laboratory determinations at baseline and day 5 for both treatment groups.


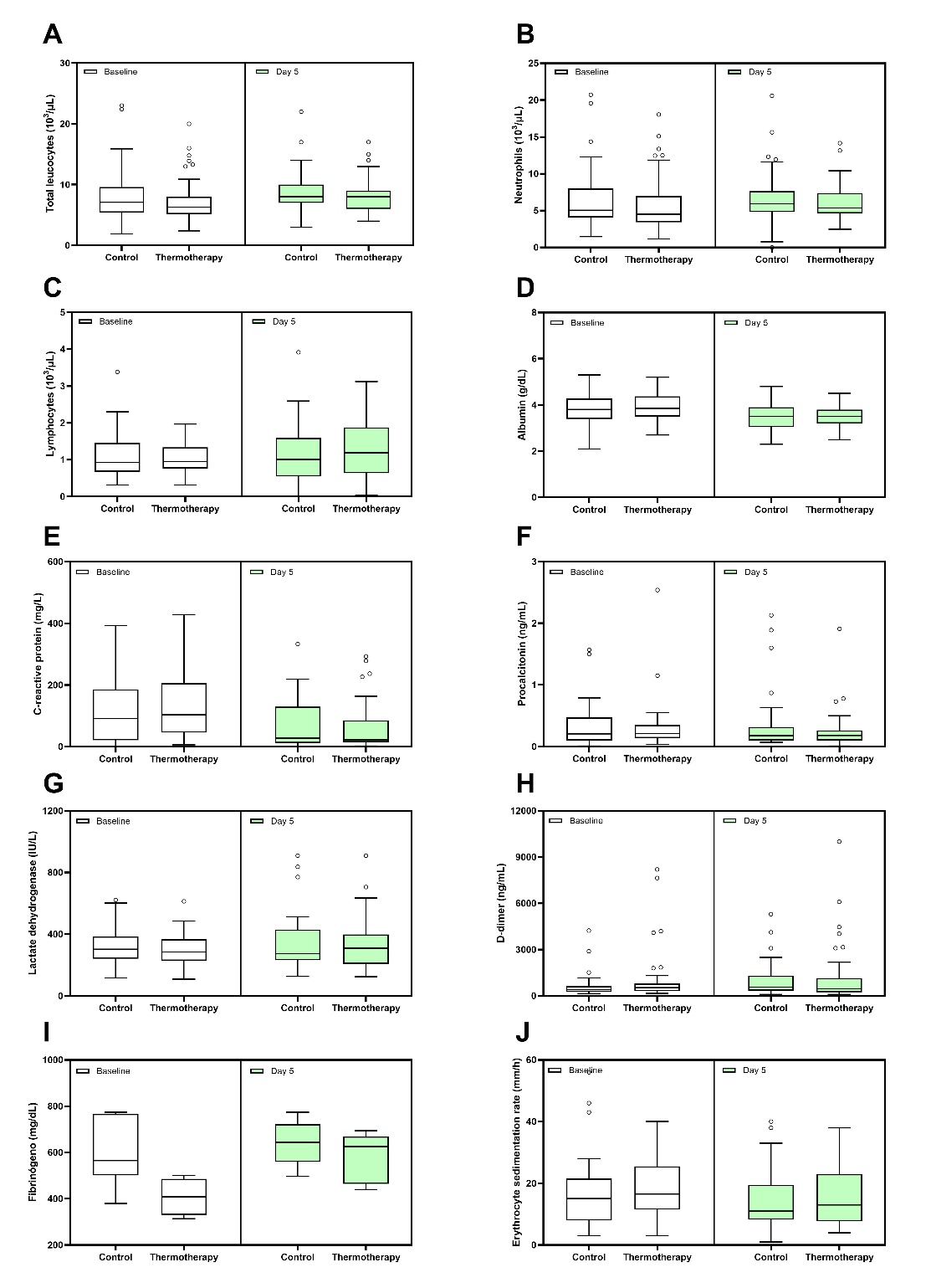


A: Total leucocytes, B: neutrophils, C: lymphocytes, D: albumin, E: C-reactive protein, F: procalcitonin, G: LDH, H: D-dimer, I: fibrinogen, J: erythrocyte sedimentation rate.

Data is presented as median, quartile 1, quartile 3 (Box), 1.5 times the inter quartile range (whiskers). Dots represent outliers.
